# A model to secure a stable iodine concentration in milk

**DOI:** 10.3402/fnr.v59.29829

**Published:** 2015-12-18

**Authors:** Gisken Trøan, Lisbeth Dahl, Helle Margrete Meltzer, Marianne Hope Abel, Ulf Geir Indahl, Anna Haug, Egil Prestløkken

**Affiliations:** 1 Department of Animal and Aquacultural Sciences, Norwegian University of Life Sciences, Ås, Norway; 2 National Institute of Nutrition and Seafood Research (NIFES), Bergen, Norway; 3 Division of Environmental Medicine, Norwegian Institute of Public Health, Oslo, Norway; 4 Tine SA, Oslo, Norway; 5 Department of Mathematical Sciences and Technology, Norwegian University of Life Sciences, Ås, Norway

**Keywords:** iodine, milk, rapeseed, fodder, model, diet

## Abstract

**Background:**

Dairy products account for approximately 60% of the iodine intake in the Norwegian population. The iodine concentration in cow's milk varies considerably, depending on feeding practices, season, and amount of iodine and rapeseed products in cow fodder. The variation in iodine in milk affects the risk of iodine deficiency or excess in the population.

**Objective:**

The first goal of this study was to develop a model to predict the iodine concentration in milk based on the concentration of iodine and rapeseed or glucosinolate in feed, as a tool to securing stable iodine concentration in milk. A second aim was to estimate the impact of different iodine levels in milk on iodine nutrition in the Norwegian population.

**Design:**

Two models were developed on the basis of results from eight published and two unpublished studies from the past 20 years. The models were based on different iodine concentrations in the fodder combined with either glucosinolate (Model 1) or rapeseed cake/meal (Model 2). To illustrate the impact of different iodine concentrations in milk on iodine intake, we simulated the iodine contribution from dairy products in different population groups based on food intake data in the most recent dietary surveys in Norway.

**Results:**

The models developed could predict iodine concentration in milk. Cross-validation showed good fit and confirmed the explanatory power of the models. Our calculations showed that dairy products with current iodine level in milk (200 µg/kg) cover 68, 49, 108 and 56% of the daily iodine requirements for men, women, 2-year-old children, and pregnant women, respectively.

**Conclusions:**

Securing a stable level of iodine in milk by adjusting iodine concentration in different cow feeds is thus important for preventing excess intake in small children and iodine deficiency in pregnant and non-pregnant women.

Milk and dairy products are currently the most important iodine sources in the Norwegian diet, contributing on average 55–70% of the daily iodine intake (1, 2). With few other significant dietary iodine sources, a stable level of iodine in the milk ensures adequate levels of iodine in the population. Prior to 1950, the iodine deficiency disorder goiter was widespread, especially in inland areas of Norway, because of low fish intake (3). Since the 1950s, iodine has been added to cow feed (4), and the high transfer (11–56%) of iodine from feed to milk (5), combined with the high consumption of dairy products, has made milk the primary source of iodine for Norwegians (1, 3, 6). As a result, clinical signs of iodine deficiency have been near absent since the 1950s and Norwegians have been considered one of the few iodine-replete populations in Europe.

Despite the fact that the concentration of iodine added to cattle feed has remained unchanged for many years (2 mg I/kg), the concentration of iodine in milk varies considerably depending on geographical location, year, and season (7–9). The iodine concentration in Norwegian summer milk has been measured from 1971 to 2008, at 66±8.5, 93±17, and 92±34 µg/kg respectively (7–9). Different from the concentration of iodine in winter milk which fell from 231±34 µg/kg in 2000, according to measurements in samples from 19 different dairies (9), to 122±40 µg/kg in 2008 from 19 different milk tour samples (7). The main hypothesis is that the sizable iodine reduction in winter milk is related to the rise in the use of new rapeseed products in cow fodder in recent years. In 2008, when the study of Haug et al. (7) was conducted, 83,415 metric tons of rapeseed products were imported, compared with a mere 2,396 tons in 2000 (10). Most of these imported rapeseed products were used in dairy cow feed. To compensate for the reduction in iodine concenof 2011 increased iodine fortification in concentrate feed from 2.0 to 3.5 mg/kg. The most recent analyses of milk from Norway's largest dairy company (TINE) from 2012 to 2013, collected at different dairies all over Norway, show 212±78 and 128±92 µg I/kg for winter and summer milk, respectively (TINE SA, unpublished data). TINE's iodine analyses confirm the findings of Dahl et al. (9) and Haug et al. (7), that is, that iodine concentration in milk is highly variable and is lower in the summer season, probably because of the fact that cows are on pasture and consequently ingest less iodine-fortified concentrate (7, 11, 12).

The iodine concentration in cow's milk depends on several factors, such as iodine intake, amount of goitrogens in the rations, application of teat dipping containing iodine, iodine source, lactation stage, milk yield, and milk processing (5, 13–16). The most important factor affecting iodine concentration is iodine intake (5), but several studies have shown that the use of rapeseed significantly reduces milk iodine levels in dairy cows (5, 17–20). Rapeseed contains glucosinolates (GSL) that are degraded to thiocyanate by the enzyme myrosinase. The thiocyanate ion (SCN^−^) competitively inhibits iodine uptake to the mammary gland (21–23).

Models for a theoretical prediction of iodine concentration in milk have been introduced; however, these models have used iodine intake as the only variable (24–26) and do little to explain variations in iodine concentration. Moreover, they indicate that other factors, such as goitrogenic substances, considerably affect milk iodine concentration. Franke et al. (5) developed separate equations to predict iodine concentration in milk, both with and without rapeseed in the cows’ diet, and these models explained more than 90% of the variation. To our knowledge, no model has included both the intake of iodine and the intake of rapeseed products, or the intake of GSL, to predict iodine concentrations in milk.

Until recently the population of Norway was assumed to have an adequate iodine intake. However, studies published during the past decade suggest that subgroups of the population are at risk of mild-to-moderate iodine deficiency (1, 2). Vulnerable groups include pregnant women, who have a higher need for iodine (2), adolescents, who have low average milk consumption (1), and a growing group of people who avoid or limit their intake of dairy products (27). From 1979 to 2014, the mean consumption of milk has decreased from 186 to 90 kg/person per year (28).

In this study, the goal was to develop a model to predict iodine concentrations in milk produced with varying levels of iodine, rapeseed, and GSL in the feed, and to evaluate the reliability of the model as a tool to ensure less variation in the iodine concentration in milk. A second aim was to estimate the impact of different iodine levels in milk on iodine nutrition in children, adults, and pregnant women in Norway.

## Material and methods

### Dataset and selection criteria

A literature review was conducted in August 2015 to track feeding experiments with dairy cows where one had measured iodine concentration in milk at different iodine and/or rapeseed/GSL intakes. The search terms were iodine, milk, rapeseed, bovine, and cow, and the search was conducted through the computerized databases Web of Science (www.isiwebofknowledge.com/), PubMed (www.ncbi.nlm.nih.gov/pubmed), and Google scholar (www.scholar.google.no/). The search resulted in 17 published studies from 1973 to 2015. The following selection criteria were used to select eligible studies: 1) Measured iodine concentration in milk, 2) Iodine intake between 0 and 5 mg I/kg dry matter (DM) feed, 3) Data on protein source and intake (with or without rapeseed meal/cake) and 4) Glucosinolate intake reported. In an attempt to represent the most up-to-date information available, experiments were only considered eligible if published in the last 20 years (since 1995). The studies from 1973 to 1993 had a much higher GSL intake and lower iodine intake compared with more recent studies and current feeding practices. The iodine analyses have also improved over the past few years, and one can therefore assume that more recent iodine measurements in milk are more accurate. In total, eight studies from 1995 to 2015 (5, 29–35) and two unpublished studies (Trøan et al., unpublished data), comprising 69 treatment means, were used to develop two models to predict iodine concentration in milk (µg/kg). Iodine was measured using Sandell–Kolthoff, ion-sensitive electrode, and colorimetric microplate methods in one study each and was measured using inductively coupled plasma mass spectrometer in seven studies.

### Statistical analysis

A compilation of the available data from the literature study resulted in a dataset containing the 69 aforementioned treatment means. No outliers were detected. Iodine concentration in milk is a strict positive quantity. The response variable was therefore log-transformed before fitting the models included in our statistical analysis. As our dataset included 10 different studies, the statistical analysis was started by exclusively investigating potential effects of studies (considered as nominal variable) on the response variable (the log-transformed iodine concentration in milk).

The model used,

log(iodine concentration)~1+studies

showed no significant effect of studies on the response variable (*p*-values were in the range 0.18–0.81). The *p*-value of the associated *F*-statistic was 0.81, and the corresponding adjusted *R*-Squared was −0.0265. Consequently, the effect of studies was excluded as a variable from the subsequent analysis.

A stepwise analysis by the fitlm-function in MATLAB^®^ R2014a, including possible interactions and higher order terms (up to order 3), resulted in two models for the prediction of iodine concentration in milk. All variables and higher order terms and interactions included in the models are supported by a *p*<0.05. The variables included in the models were:

1. Iodine intake (mg I/kg DM), denoted *iodineIn*
2. Intake of rapeseed meal or cake (grapeseed product/kg DM), denoted *rapeseed*
3. Glucosinolate intake (mmol/day), denoted *gsl*

The two models are presented in Tables 1 and 2. In Model 1, *gsl* was included as the independent variable in addition to iodine, whereas in Model 2, *rapeseed* was included as the independent variable. When including both *gsl* and *rapeseed* in the stepwise modeling, only one of them proved to be significant (*p*<0.05) and no model including both *gls* and *rapeseed* is thus presented. Both models were evaluated through ‘leave-one-out’ cross-validation, confirming good predictive stability.

**Table 1:** Parameters of the model analysis developed by the fitlm-function in MATLAB^®^ R2014a

Parameters^a^	Estimate	SE	CI	*p*
Intercept	4.16	0.218	3.72 to 4.59	<0.001
IodineIn	1.89	0.343	1.21 to 2.58	<0.001
Gsl	−0.152	0.031	−0.21 to −0.09	<0.001
IodineIn:Gsl	0.023	0.011	0.00075 to –0.045	0.043
IodineIn^2	−0.599	0.16	−0.91 to −0.28	<0.001
Gsl^2	0.0069	0.0015	0.0038 to 0.0099	<0.001
IodineIn:Gsl^2	−0.0016	0.00051	−0.0026 to −0.00053	0.0035
IodineIn^3	0.069	0.02	0.029 to 0.11	0.0012
Gsl^3	−4.31e-05	9.24e-06	−6.16e-05 to −2.47e-05	<0.001

Number of observations: 69, Error degrees of freedom: 60.

a IodineIn=Iodine intake (mg I/kg DM); Gsl=Glucosinolates intake (mmol/day).

^2 and ^3 indicate second and third order terms, respectively.

**Table 2:** Parameters of the developed model analysis by the fitlm-function in MATLAB^®^ R2014a

Parameters^a^	Estimate	SE	CI	*p*
Intercept	4.22	0.184	3.85 to 4.59	<0.001
IodineIn	1.84	0.303	1.24 to 2.45	<0.001
Rapeseed	−0.01	0.002	−0.015 to −0.0057	<0.001
IodineIn^2	−0.57	0.141	−0.85 to −0.29	<0.001
Rapeseed^2	2.87e-05	1.28e-05	3.09e-06 to 5.43e-05	0.029
IodineIn^3	0.07	0.018	0.03 to 0.10	<0.001

Number of observations: 69, Error degrees of freedom: 63.

a IodineIn=Iodine intake (mg I/kg DM) and Rapeseed=Intake of rapeseed meal or cake (g rapeseed product/kg DM).

^2 and ^3 indicate second and third order terms, respectively.

### Calculation of iodine intake from milk and dairy products

The following surveys have been used to estimate the intake of dairy products in the Norwegian population – in adults: ‘Norkost 3’ (36), in 2-year-old children: ‘Småbarnskost’, (37) and in pregnant women: the Norwegian Mother and Child Cohort Study (MoBa) (38). ‘Norkost 3’ is a dietary survey of adult Norwegians conducted during the years 2010–2011, including 862 men and 925 women aged between 18 and 70 years, combining repeated 24-h food recall telephone interviews with a food frequency questionnaire (36). ‘Småbarnskost’ includes data from 1,674 2-year-old children in 2007 and is based on a food frequency questionnaire (37). In addition, data from 11,664 pregnant women participating in MoBa in 2008 and who had filled out a food frequency questionnaire in mid-pregnancy were included (38).

On the basis of data in the Norwegian food composition table (39), on-pack nutrition information and knowledge of consumption of different dairy products within the groups, values for iodine concentrations in different dairy product groups were estimated. Mean iodine intake from milk and dairy products was then calculated for men, women, 2-year-olds, and pregnant women based on their daily intake (g/day) of white milk (pasteurized/cultured), flavored milk (pasteurized/cultured), yoghurt, cheese, whey cheese, and cream products (Table 3). We also calculated how much the iodine intake from dairy products contributed to daily requirements of men, women, 2-year-old children, and pregnant women. The results were used to simulate how varying cow fodder compositions would affect iodine intake from dairy products in selected populations.

**Table 3:** The average intake of dairy products by men, women, 2-year-old children and pregnant women; the iodine concentration in these products, and daily iodine intake from dairy products in these four groups

	Average intake^a^					Iodine intake from dairy products			
			
	g/day				Iodine conc.^b^	µg/day			
		
	Men *n*=862	Women *n*=925	2-year-old children *n*=1,674	Pregnant *n*=11,664	µg/100 g	Men *n*=862	Women *n*=925	2-year-old children *n*=1,674	Pregnant *n*=11,664
Milk^c^	340	204	316	308	20	68	41	63	62
Milk^d^	21	13	4	43	16	3	2	1	7
Yoghurt	23	32	71	67	19	4	6	13	13
Cheese	40	36	12	16	35	14	13	4	6
Whey cheese	6	6	10	6	140	8	8	14	9
Cream products	22	21	8	17	19	4	4	2	3
Total iodine intake from dairy products (µg/day)						102	74	97	99
% iodine of recommended intake^e^						68	49	108	56

a Food intake data from ‘Norkost 3’ survey (36), ‘Småbarnskost’ survey (37), and the Norwegian Mother and Child Cohort Study of pregnant women from 2008 (38).

b An estimated representative iodine concentration based on data in the Norwegian food composition table (39).

c Milk (pasteurized/cultured).

d Flavored milk (pasteurized/cultured).

e Recommended intake of iodine for adults are 150 µg/day, 2-year old children 90 µg/day and pregnant women 175 µg/day (47).

## Results

### Model development and evaluation

The two models developed for predicting iodine concentration in milk are presented below. Model 1 developed for predicting iodine concentration in milk with GSL intake is:

$$\text{log(iodine concentration)}\sim \text{1+iodineIn}+\text{gsl}+\text{iodineIn:gsl+}{\text{iodineIn}}^{2}\text{+}{\text{gsl}}^{\text{2}}\text{+iodineIn:gs}{\text{l}}^{2}+\text{iodineI}{\text{n}}^{\text{3}}+\text{gs}{\text{l}}^{\text{3}}$$

The estimated parameters in Model 1 are presented in Table 1. The Root Mean Squared Error (RMSE) was 0.416 and the adjusted R-Squared 0.79. The Root Mean Square Error of Cross-Validation (RMSECV) was 0.427.

Model 2 developed for predicting iodine concentration in milk with rapeseed intake is:

$$\text{log(iodine concentration)}\sim \text{1+iodineIn+rapeseed+iodineI}{\text{n}}^{\text{2}}+\text{rapesee}{\text{d}}^{\text{2}}+\text{iodineI}{\text{n}}^{\text{3}}$$

The estimated parameters in Model 2 are presented in Table 2. The RMSE was 0.382 and the adjusted R-Squared 0.83. The RMSECV was 0.393.

**Fig. 1:**
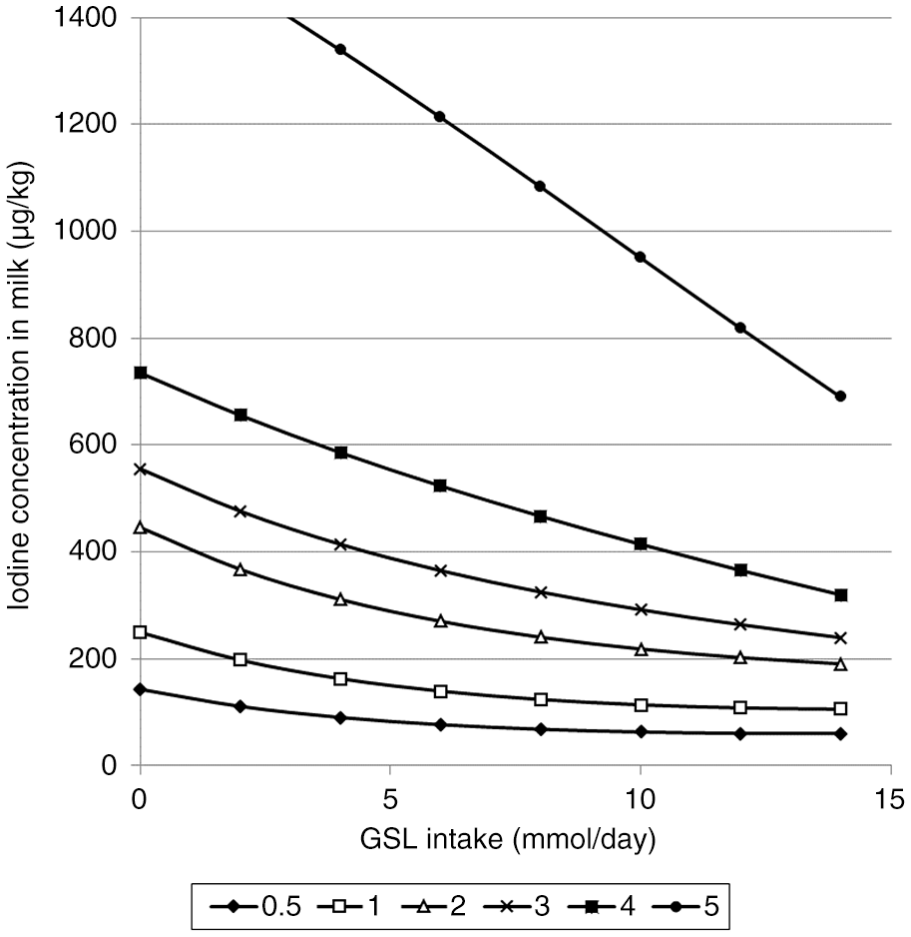
The average predicted iodine concentration in milk (µg/kg) from Model 1 at iodine intake of 0.5, 1, 2, 3, 4, and 5 mg I/kg DM and glucosinolate intake of 0–14 mmol/day.

**Fig. 2:**
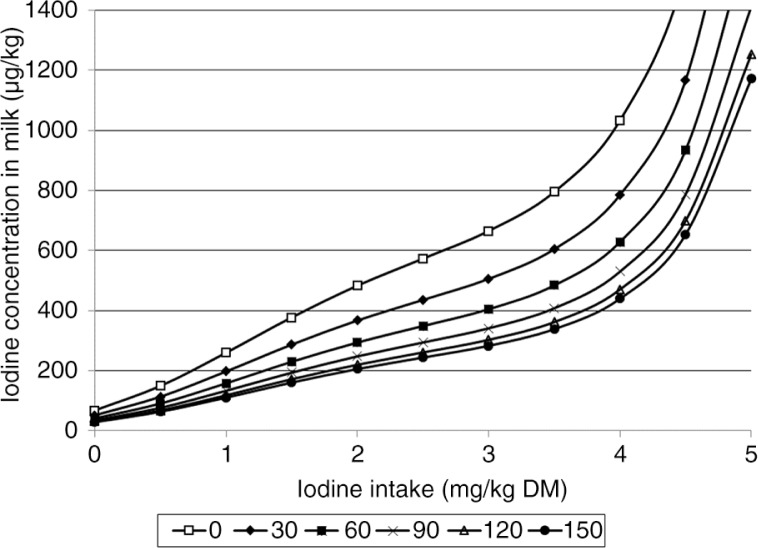
The average predicted iodine concentration in milk (µg/kg) from Model 2 at rapeseed cake or meal intake of 0, 30, 60, 90, 120, and 150 g/kg DM and iodine intake of 0–5 mg I/kg DM.

The predicted iodine concentrations of milk from Models 1 and 2 (Tables 1 and 2) developed for different iodine and GSL or rapeseed intakes are presented in Figs. 1 and 2.

### Iodine intake from milk and dairy products in the population groups

The daily intake of dairy products, iodine concentration in the dairy products, and the estimated mean iodine intake from dairy products for men, women, 2-year-old children, and pregnant women are presented in Table 3.

### Dietary impact of varying iodine concentration in Norwegian milk

**Fig. 3:**
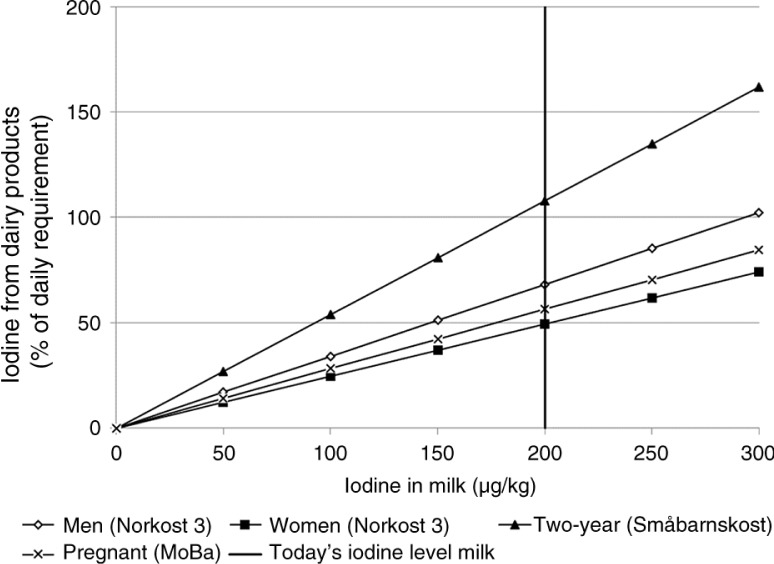
Estimated iodine intake from dairy products (in percentage of recommended intake, NNR (47) in men, women, 2-year-old children, and pregnant women with different iodine concentrations in milk (µg/kg). Today's iodine level in milk of 200 µg/kg, is indicated by a vertical line.

With varying iodine concentration in milk, the iodine intake from dairy products of the four studied population groups is stipulated as shown in Fig. 3.

## Discussion

In this study, we developed two models (Tables 1 and 2) to predict iodine concentration in milk based on iodine and GSL or rapeseed content in dairy cow feed. The new models help explain the variation in milk iodine concentration and can be used to determine the optimal amount of iodine to add to feeds for dairy cows at varying levels of rapeseed products or GSLs in the diet. Further, our simulation results suggest that ensuring a stable level of iodine in milk is important to avoid iodine deficiency and/or excess intake of iodine in the Norwegian population. The intake of iodine from dairy products in the four selected population groups showed that, with a milk iodine concentration of 200 µg/kg, dairy products today cover on average from 49 to 108% of the recommended iodine intake in different groups of the population and that most of the iodine intake from dairy products is from milk (Table 3).

### Model development and evaluation

The presented models can be used to predict iodine concentration in milk for herds or groups of cows but not for individual cows. In the study by Franke et al. (15), 7–12% of the variation in iodine content in the milk was explained by differences among animals and 51–63% by lactation stage. In the models developed in the present study, which were based on group averages, this variation has already been evened out. Model 1 included more estimated parameters and got a *R* ^2^ of 79%. In Model 2 (Table 2), the variables *iodineIn, rapeseed*, and their higher order terms explained 83% of the log-transformed iodine concentration in milk. However, since the RMSE and RMSECV values were similar, the cross-validation of both models showed a good fit for the predicted values. Moreover, the predicted iodine concentrations in milk for both models were quite similar (Figs. 1 and 2). Thus, depending on the available data, the better suited of the two models can be selected to predict milk iodine concentration.

As expected, Model 2 predicted lower milk iodine concentration with greater concentration of rapeseed in the diet. However, when the concentration of rapeseed exceeds approx. 180 g/kg DM, the model begins to predict increased iodine concentration in milk. This is not logical and we find no physiological explanation for this. A probable explanation can be found in the positive second order term of rapeseed in the model. The highest amount of rapeseed product included in the studies in Model 2 is 236 g/kg DM, and the model does therefore not predict correct milk iodine concentration at high intakes of rapeseed product. Accordingly, Model 2 is only valid for rapeseed cake or rapeseed meal intakes from 0 to 180 g/kg DM. The same type of limitation exists for Model 1 with *gsl*. This model is only valid up to a GSL intake of 14 mmol/day. With respect to iodine intake, the models are close to linear and predict milk iodine concentration precisely up to 3.5–4.0 mg I/kg DM (Figs. 1 and 2). However, for iodine intakes above 3.5–4.0 mg/kg DM, the models begin to predict unrealistically high iodine concentrations in milk (Figs. 1 and 2). Schone et al. (32) reported an iodine concentration in milk of 2,750±852 µg/kg with an iodine intake of 10.1 mg/kg DM and no rapeseed products in the fodder. This is in the same range as that predicted by Model 2 with an intake of 5 mg I/kg DM. The datasets used to develop our models contain few data with an iodine intake above 4 mg/kg DM. Therefore, both models have uncertain estimates above this level and are only valid for iodine intake from 0 to 4 mg/kg DM. Previous studies have indicated that the iodine uptake of the mammary gland is saturated if iodine intake exceeds 1,000 mg/day (40, 41), which is far above the highest iodine intake used in the development of models in this study.

Several studies have shown that including rapeseed cake or rapeseed meal in the feed reduces milk iodine concentration. This is explained by GSLs and their breakdown products (e.g. thiocyanate) (5, 17, 20). However, high or low GSL intake seems to be of minor importance, as the differences found in iodine and thiocyanate concentrations in milk between different levels of GSL intake were low (18, 30). However, there are considerable differences in milk iodine levels when feeds with and without rapeseed products are offered (5, 30). In contrast to previous models (5, 24–26), the models in the present study included intake of GSL or rapeseed products in addition to iodine as variables in the same model. The model explains a large part of the variation in milk iodine concentration. A strength of our models, in addition to including the *rapeseed* or the *gls* variable, is that they are based on data from 10 independent studies and include data from previously developed models.

In Norway, 10% rapeseed cake containing 10% fat is commonly included in the concentrate, giving approximately 50–70 g/kg DM rapeseed cake in the total diet (Felleskjøpet Fôrutvikling, Trondheim, Norway, personal communication). Extracted rapeseed meal with low fat content is used at levels up to 20–25% of the concentrate (corresponding to approx. 100 g/kg DM in the total ration), which is in the valid range of Model 2. Although dependent on mineral oil prices, it is likely that the production of biofuel from oilseeds will continue and probably increase in the coming years. Ruminants are the most efficient handlers of rapeseed residues originating from such production. Thus, the challenge is not to exclude these products in ruminant diets but to find efficient ways of using them without compromising the concentration of iodine in milk. Hermansen et al. (30) concluded that a maximal effect of rapeseed products on reduction of iodine in milk was achieved at an intake of less than 50 mmol GSL/day or when the total diet contained 106–110 g/kg DM of double-low rapeseed cake or rapeseed meal. From Model 2, it would appear that the threshold concentration of iodine in milk is not achieved before rapeseed intake is 120–150 g/kg DM of feed (Fig. 2. However, the average GSL intake from the studies used to develop the model was 20±24.1 mmol/day, and only two of the studies had a GSL intake above 50 mmol/day (29, 34). Nevertheless, the models presented indicate that when controlled, rapeseed products can be included in dairy cow diets in fairly high amounts.

### Variation in iodine concentration in milk depending on feeding practice

Today, the maximum iodine concentration allowed in feed rations is 5 mg/kg DM (42). However, EFSA is discussing lowering the maximum level to 2 mg I/kg DM (43), out of concern that human overconsumption of iodine can be attributed to high iodine concentration in milk in countries that also have a high level of iodine in iodine-fortified salt (43). The median iodine concentration in cultivated grass for silage is only 0.5 mg/kg DM on average (range <0.2–3.5 mg I/kg DM depending on geographical location) (44). Thus, the main source of iodine of Norwegian dairy cows today is the 3.5 mg I/kg DM in the form of calcium iodate added to concentrate feed. The daily ration for dairy cows usually consists of silage and concentrate, and one may assume an iodine intake of 1.5–2 mg/kg DM, corresponding to a total of 30–50 mg/day. However, commercial mineral supplements could contribute 10–40 mg I/day. Moreover, teat dipping with disinfectant containing iodine also affects milk iodine content. Teat dipping with iodine is not commonly used in Norway, but it is recommended for mastitis infection with *Streptococcus dysgalactiae* (45). When used, the iodine level in the disinfectant seems to be more important for the transfer of iodine to milk than time of dipping. Spraying with a 1% iodine solution has resulted in a doubling of the iodine content in milk (14, 46). Thus, depending on feeding practice, the concentrate:silage ratio, the amount of mineral supplement offered, the practice of teat dipping/spraying and geographical location of the farm, the iodine intake of the cow, and consequently iodine concentration in the milk may vary considerably. However, it is important to emphasize that the majority of Norwegian dairy cows are fed a diet consisting of grass silage and a commercial iodine-fortified concentrated feed only, thus allowing for production of milk with stable iodine content.

### Iodine intake from dairy products

There are few other iodine sources in the Norwegian diet, and unless seafood intake is high or iodine-containing supplements are used, one is dependent on dairy products to cover the daily iodine intake. In the four investigated population groups in this study, milk and dairy products are major contributors to iodine intake (Table 3). This is in line with earlier studies (1, 2). The share of milk and dairy products are, however, highly variable between individuals and population groups. On average, women have the lowest iodine intake from dairy products, which only covers an estimated 49% of women's daily recommended intake, while 2-year-old children ingest more than the recommended level (90 µg) from dairy products alone (Table 3). From data on adults collected 1997–2000, Dahl et al. (1) concluded that the iodine status in subgroups of the population was low and identified people with a low dairy product intake as one of the groups at risk of iodine deficiency. Twenty five percent of women in ‘Norkost 3’ had an intake of milk and yoghurt of less than 75 g/day (36), indicating that there is considerable variation in iodine intake in the population. Of the dairy products, milk contributed the largest share (58–69%), while cheese only contributed 4–18% of total iodine intake from dairy products (Table 3). The consumption of milk has decreased since the 1980s; however, in the same time period, the consumption of cheese has increased (28). In the survey ‘Spisefakta 2009’, representative of the Norwegian adult population, 18% (23% of women and 14% of men) recorded that they regularly or sometimes avoided dairy products (27). Therefore, although dairy products contribute significantly to the intake of iodine in the average Norwegian diet, the contribution varies greatly between individuals and population groups. Data from the MoBa Study showed that 54% of pregnant women in the study had an iodine intake below the recommended 175 µg I/day (47), and 78% had an intake below 250 µg I/day, the level recommended by the World Health Organization (2, 48). One of the main reasons for the low iodine intake was low consumption of milk and dairy products. Twenty eight percent of the pregnant women consumed less than 200 mL/day of milk and yoghurt, putting them at high risk of having an iodine intake associated with mild-to-moderate iodine deficiency (2). Similar concerns have been described in the United Kingdom and Iceland (49, 50). From a British study of pregnant women and their children, mild and moderate iodine deficiency during pregnancy was associated with adverse long-term effects on the children's cognitive outcomes (50).

The 2-year-old children, and especially those consuming large amounts of milk, are at risk of excess daily intake of iodine, even with current iodine levels in milk. For children between 1 and 3 years, the upper tolerance level for iodine has been set at 200 µg/day (51); with today's iodine level in milk, the average 2-year-old will have a daily iodine intake of about 97 µg/day from dairy products alone (Fig. 2). From the nationally representative study ‘Småbarnskost’, the 95th percentile of 2-year-olds consumed 776 g/day of all types of milk (37), resulting in about 150 µg/day from milk alone at today's iodine concentration in milk of 200 µg/kg. In the event of higher concentrations of iodine in milk, many 2-year-old children are at risk of excess intake of iodine, above the toddler's tolerable intake level. The optimal iodine level in milk thus seems to lie within a relatively narrow interval (52). An iodine intake below or above the recommended level is associated with an increased risk of thyroid diseases in the population (52).

### How can we secure a stable concentration of iodine in Norwegian milk?

The above discussion underlines the importance of minimizing the variation of iodine in milk. To achieve this, the iodine intake in cows needs to be controlled carefully. With today's level of iodine and rapeseed products in cow feed, the predicted iodine concentration in milk from Model 2 (Fig. 2) is between 216 and 315 µg I/kg. The predicted values are higher than the iodine concentration found in analyses of milk today, with a weighted average of 200 µg I/kg (TINE, unpublished data). However, behind the weighted average, there will be herds being given iodine and rapeseed that are both above and below the current estimated level. In concentrates without rapeseed products, the iodine concentration needs to be lower than in concentrates containing rapeseed products. EFSA, based on the study of Franke et al. (5), suggests iodine supplementation for dairy cows of 1 mg I/kg DM diet with a GSL-free diet and 2 mg I/kg DM diet with a diet containing GSL, giving 250 and 200 µg I/kg milk, respectively (43). Our data corresponds well with this (Fig. 2). According to Model 2, with an iodine intake of 1 mg/kg DM and no rapeseed in the diet, the predicted iodine concentration in milk is 260 µg I/kg, compared with 206 µg I/kg in milk for an iodine intake of 2 mg I/kg DM and a diet containing 150 g rapeseed product/kg DM (Fig. 2). With the concentrate:silage ratio of 40:60 (53) and current levels of iodine in the concentrate, there is a risk that the milk will contain iodine concentrations that are too high if the cow is also supplemented with iodine from mineral premixes or if teats are dipped or sprayed with disinfectants containing iodine. In addition, the opposite problem occurs if the cow's diet is based mostly on silage with little supplementation of concentrate or mineral premixes containing iodine, as this could lead to low iodine concentrations in milk.

This study shows that milk and dairy products are major sources of iodine in the Norwegian diet and that keeping iodine concentration in milk stable throughout the year thus is important. A reduction in iodine concentration in milk entails a greater risk of iodine intake below the recommended levels for the population. This applies particularly to women, including pregnant women. However, higher iodine concentration in milk will increase the risk of excess iodine intake in small children who drink more than 3 dl of milk per day. Iodine concentrations in milk need to be monitored closely. The models developed in the present work can be used to adjust cow iodine intake so that the iodine concentration in milk remains approximately the same throughout the year.
